# Does Soluble TREM2 Protect Against Alzheimer's Disease?

**DOI:** 10.3389/fnagi.2021.834697

**Published:** 2022-01-28

**Authors:** Guy C. Brown, Peter St George-Hyslop

**Affiliations:** ^1^Department of Biochemistry, University of Cambridge, Cambridge, United Kingdom; ^2^Department of Medicine, University of Cambridge, Cambridge, United Kingdom; ^3^Department of Medicine, University of Toronto, Toronto, ON, Canada

**Keywords:** TREM2, sTREM2, microglia, Alzheimer's disease, amyloid beta, neuroinflammation, neurodegeneration, neuroprotection

## Abstract

Triggering Receptor Expressed in Myeloid Cells 2 (TREM2) is a pattern recognition receptor on myeloid cells, and is upregulated on microglia surrounding amyloid plaques in Alzheimer's disease (AD). Rare, heterozygous mutations in TREM2 (e.g., R47H) increase AD risk several fold. TREM2 can be cleaved at the plasma membrane by metalloproteases to release the ectodomain as soluble TREM2 (sTREM2). Wild-type sTREM2 binds oligomeric amyloid beta (Aβ) and acts as an extracellular chaperone, blocking and reversing Aβ oligomerization and fibrillization, and preventing Aβ-induced neuronal loss *in vitro*. Whereas, R47H sTREM2 increases Aβ fibrillization and neurotoxicity. AD brains expressing R47H TREM2 have more fibrous plaques with more neuritic pathology around these plaques, consistent with R47H sTREM2 promoting Aβ fibrillization relative to WT sTREM2. Brain expression or injection of wild-type sTREM2 reduces pathology in amyloid models of AD in mice, indicating that wild-type sTREM2 is protective against amyloid pathology. Levels of sTREM2 in cerebrospinal fluid (CSF) fall prior to AD, rise in early AD, and fall again in late AD. People with higher sTREM2 levels in CSF progress more slowly into and through AD than do people with lower sTREM2 levels, suggesting that sTREM2 protects against AD. However, some of these experiments can be interpreted as full-length TREM2 protecting rather than sTREM2, and to distinguish between these two possibilities, we need more experiments testing whether sTREM2 itself protects in AD and AD models, and at what stage of disease. If sTREM2 is protective, then treatments could be designed to elevate sTREM2 in AD.

## Introduction

### TREM2

Triggering Receptor Expressed in Myeloid Cells 2 (TREM2) is a pattern recognition receptor found on the plasma membrane of myeloid cells. When activated by ligands, such as phospholipids, lipoproteins, and amyloid beta peptide (Aβ), TREM2 induces an innate immune response, which includes phagocytosis, chemotaxis, and transcriptional changes (Keren-Shaul et al., 2017; Deczkowska et al., 2020; Kulkarni et al., 2021). TREM2 signaling is mainly *via* binding DAP12 (DNAX-activating protein of 12 kDa), which activates Syk tyrosine kinase (Deczkowska et al., 2020). Within the brain, TREM2 is almost uniquely expressed by microglia, and is upregulated on microglia around amyloid plaques in AD (Giraldo et al., 2013; Yuan et al., 2016; Brendel et al., 2017). Rare, heterozygous mutations of TREM2 are known to affect AD risk, including the R47H mutation, which increases AD risk several fold (Guerreiro et al., 2012; Giraldo et al., 2013; Jonsson et al., 2013; Kulkarni et al., 2021). These mutations are thought to increase AD risk by reducing the protective roles of microglial TREM2, in particular by reducing microglial phagocytosis of amyloid plaques (Condello et al., 2015; Yuan et al., 2016).

### sTREM2

TREM2 is a single-pass type I transmembrane protein with a small C-terminal on the cytosolic side of the plasma membrane, and an N-terminal ectodomain that includes the ligand binding site (Zhong and Chen, 2019; Yang et al., 2020). However, the ectodomain of TREM2 is shed from cells expressing full-length TREM2 into the extracellular medium, and is then known as soluble TREM2 (sTREM2) (Piccio et al., 2008; Wunderlich et al., 2013). The turnover of full-length TREM2 on macrophages is very rapid with a half-life of <1 h, because of constitutive cleavage of full-length TREM2 and shedding of sTREM2 (Thornton et al., 2017). The proteases responsible for shedding sTREM2 include A Disintegrin And Metalloproteases 10 and 17 (ADAM10 and ADAM17), and this cleavage occurs at the H157-S158 peptide bond (Schlepckow et al., 2017; Thornton et al., 2017). ADAM10 and 17 appear to be responsible for sTREM2 release induced by lipopolysaccharide (LPS), whereas the protease meprin β constitutively cleaves TREM2 (predominately at the R136-D137 peptide bond) to release sTREM2 from macrophages (Berner et al., 2020). However, it is unclear whether meprin β can generate sTREM2 in microglia. After shedding of sTREM2, the remaining part of TREM2 may be cleaved within the membrane by γ secretase (Wunderlich et al., 2013). The very rapid and inducible turnover of TREM2 to generate sTREM2 suggests either that (i) TREM2 levels need to be regulated very rapidly, or (ii) that sTREM2 has a function, and full-length TREM2 is a precursor of this functional sTREM2.

### Regulation of sTREM2 Shedding

Conditions that increase or decrease sTREM2 shedding from full-length TREM2 are not clear, but LPS or IL-1β can induce sTREM2 release from primary mouse microglia (Zhong et al., 2019). Also, oligomeric Aβ, which can bind both full-length TREM2 and sTREM2, induced shedding of sTREM2 for TREM2-overexpressing cells (Vilalta et al., 2021), suggesting that sTREM2 shedding may be induced prior to and during AD as a result of Aβ oligomerization. CSF sTREM2 levels increase in amyloid mouse models and correlate with microglial activation (Brendel et al., 2017). Viral infection of the lungs can increase sTREM2 levels post-infection, due to IL-13 or IL-4 induced sTREM2 shedding (Wu et al., 2015). And HIV viral infection of the brain increases CSF levels of sTREM2 (Gisslén et al., 2018). sTREM2 levels in CSF are thought to be a biomarker of microglial activation, although there is limited evidence for this *in vivo* (Bekris et al., 2018; Rauchmann et al., 2020; Pascoal et al., 2021), and sTREM2 may itself cause microglial activation (see below). CSF sTREM2 levels rise with age in humans from about 2 ng/ml at 43 years to 6 ng/ml at 80 years of age (Henjum et al., 2016).

### Alternative Forms of sTREM2

**Figure 1:**
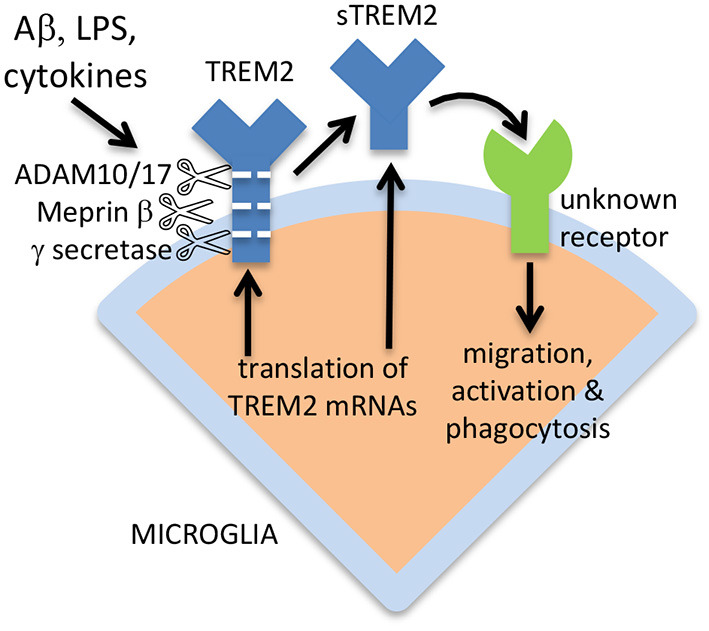
Release of sTREM2 from microglia, and activation of microglia by sTREM2. sTREM2 may be generated by ADAM10/17 or meprin β proteolysis of full-length TREM2, or from expression of an isoform lacking the transmembrane domain. γ secretase can cleave the remains of TREM2 within the membrane to degrade it. Released sTREM2 can chemoattract and activate microglia *via* unknown receptors.

*TREM2* can be expressed *via* alternative splicing as a soluble isoform, lacking the transmembrane form, and this alternative sTREM2 may constitute 25% of total sTREM2 in the brain (Ma et al., 2016; Del-Aguila et al., 2019). This again suggests that sTREM2 has a function, rather than being simply a degradation product of full-length TREM2. The sTREM2 generated by alternative splicing would be 219 amino acids residues long, the sTREM2 generated by ADAM10 or 17 would be 157 amino acids residues long, and the sTREM2 generated by meprin β would be 136 amino acids residues long (plus shorter forms) (Berner et al., 2020), although removal of the signal peptide would shorten all these sTREM2 forms by 18 amino acid residues. The ectodomain of TREM2 and sTREM2 is highly glycosylated at Asn20 and Asn79, so the apparent molecular weight of full-length TREM2 on electrophoresis gels is about 50 kDa when fully glycosylated, and about 25 kDa when deglycosylated (Ma et al., 2016). The apparent molecular weight of sTREM2 in CSF is 30–35 kDa (Ma et al., 2016), implying that almost half the apparent weight of sTREM2 is sugars, and that different glycosylation states coexist. The alternative mechanisms of sTREM2 generation are illustrated in Figure 1.

### sTREM2 Degradation

Processes responsible for degradation and clearance of extracellular sTREM2 are unclear, although it has been found that macrophages readily take up sTREM2 (Wu et al., 2015), and sTREM2 injected into mouse brain is cleared from the brain within 3 days (Zhong et al., 2019). Membrane-attached meprin β generates sTREM2 constitutively, but inflammation-induced ADAM10/17 releases soluble meprin β, which can rapidly degrade sTREM2 (Berner et al., 2020). However, it is unclear whether meprin β contributes to sTREM2 production or degradation in the brain.

## Actions of sTREM2

### sTREM2 Activates Microglia

sTREM2 treatment of macrophages induced phosphorylation of ERK1/2 (extracellular signal-regulated kinases 1 and 2) and inhibited apoptosis (Wu et al., 2015). Similarly, sTREM2 treatment of microglia in culture promoted survival by inhibiting apoptosis, apparently *via* activation of Akt (Zhong et al., 2017). In addition, sTREM2 induced inflammatory activation of cultured microglia *via* nuclear factor-κB, resulting in morphological activation and release of pro-inflammatory cytokines (Zhong et al., 2017). sTREM2 also stimulated migration and phagocytosis by primary microglia in culture (Zhong et al., 2019). Injection of sTREM2 into the brains of mice expressing the amyloid precursor protein (APP) induced activation and proliferation of microglia, plus increased expression of pro-inflammatory cytokines, and increased microglial phagocytosis of Aβ (Zhong et al., 2019). Injection of sTREM2 into the brains of healthy mice also induced expression of pro-inflammatory cytokines (Fassler et al., 2021). A fragment of sTREM2 (amino acids 51–81) was sufficient to activate microglia (Sheng et al., 2021). Thus, sTREM2 activates microglia, although the mechanism of this activation is unclear.

### sTREM2 Blocks Aβ Aggregation and Neurotoxicity

**Figure 2:**
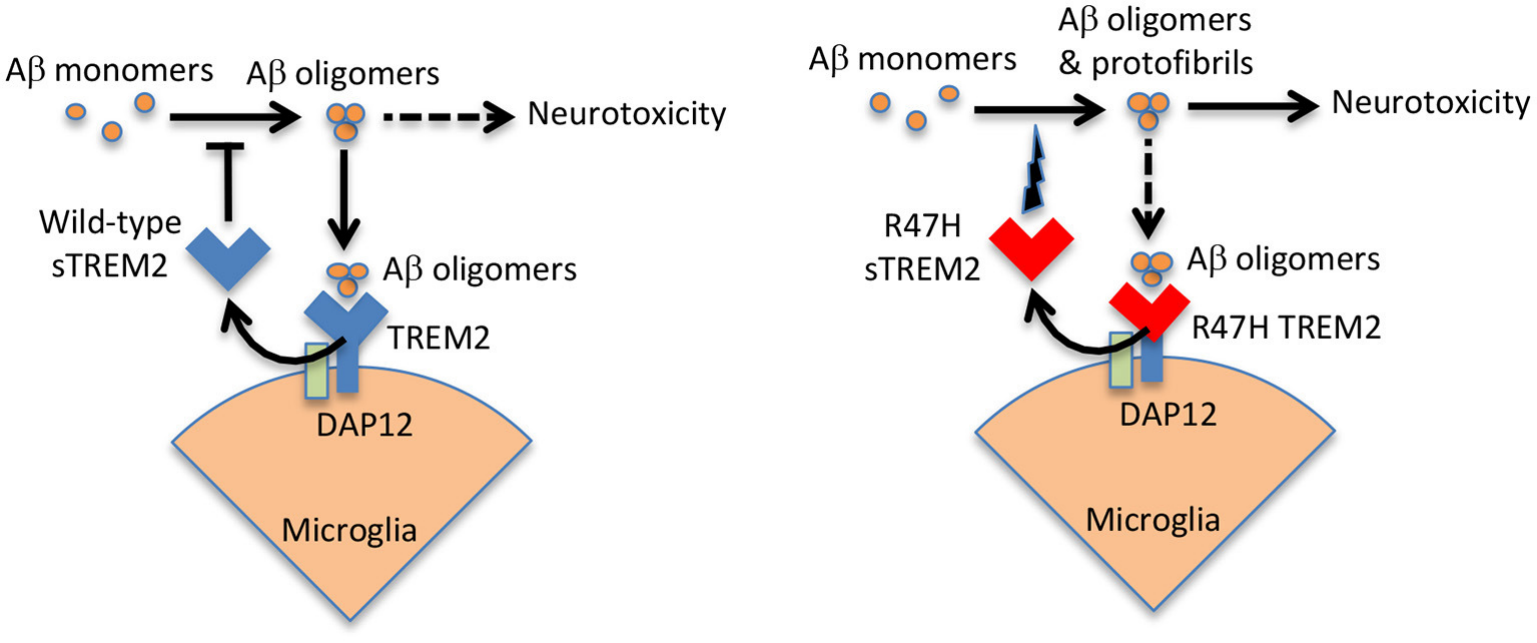
Wild-type sTREM2 blocks Aβ pathology, but R47H TREM2 does the opposite. Aβ oligomers bind to TREM2 and induce shedding of sTREM2. Wild-type sTREM2 blocks Aβ oligomerization, fibrillization and neurotoxicity. R47H sTREM2 increases Aβ oligomerization, fibrillization and neurotoxicity. Thus, wild-type sTREM2 may protect against amyloid pathology, while R47H TREM2 exacerbates amyloid pathology. This might help explain why a single copy of the R47H TREM2 gene increases AD risk several fold.

sTREM2 is known to bind oligomeric Aβ, with minimal binding to monomeric or fibrillar Aβ (Lessard et al., 2018; Zhao et al., 2018; Zhong et al., 2018; Vilalta et al., 2021). Subsequently, it was found that sTREM2 blocked Aβ oligomerisation and fibrillization at a molar ratio of 1 sTREM2 to 100 Aβ (Kober et al., 2021; Vilalta et al., 2021), and at higher molar ratios sTREM2 disaggregated Aβ oligomers and fibrils (Vilalta et al., 2021). Wild-type sTREM2 also inhibited Aβ-induced permeabilization of artificial membranes, and inhibited Aβ-induced neuronal loss in glial-neuronal cultures (Vilalta et al., 2021). These results suggest that wild-type sTREM2 may act as extracellular chaperone for Aβ, blocking its folding into aggregatable forms and refolding aggregates into soluble forms, thereby inhibiting the neurotoxicity of Aβ. In contrast, R47H sTREM2 bound less to Aβ oligomers, but increased Aβ aggregation into protofibrils, and increased Aβ-induced neuronal loss in glial-neuronal cultures (Vilalta et al., 2021). Thus, R47H sTREM2 may not only loose a neuroprotective function, but also gain a neurotoxic function in the presence of Aβ, probably by folding Aβ into more toxic forms (see Figure 2).

### sTREM2 Protects Against Amyloid Pathology in Mice

sTREM2 injection into the brains of mice expressing APP reduced amyloid plaque load (Zhong et al., 2019). Furthermore, viral expression of sTREM2 in the APP-expressing mice, reduced plaque load and reversed deficits of spatial memory and long-term potentiation (Zhong et al., 2019). Thus, sTREM2 is protective against amyloid pathology in mice, and this might be by sTREM2 affecting Aβ aggregation and/or sTREM2 activating microglia to phagocytose plaques. A fragment of sTREM2 (amino acids 51–81) was sufficient to activate microglia, but not to bind Aβ and reduce amyloid pathology *in vivo*; whereas a 41–81 fragment of sTREM2 bound Aβ and reduced amyloid pathology *in vivo* better than full-length sTREM2 (Sheng et al., 2021). This suggests that sTREM2 protects against amyloid pathology mainly by binding Aβ.

TREM2 knockout mice, crossed with APP-expressing mice, have more fibrous and less compact plaques (Condello et al., 2015; Wang et al., 2016; Yuan et al., 2016; Song et al., 2018), and while this has been attributed to less microglial phagocytosis of the plaques because of less full-length TREM2, the result might alternatively be due to sTREM2 blocking Aβ aggregation and/or sTREM2 activating microglia to phagocytose plaques. TREM2 knockout mice have increased Aβ seeding (Parhizkar et al., 2019), which again could be explained by reduced microglial phagocytosis of Aβ seeds mediated by full-length TREM2, or reduced blocking of Aβ aggregation by sTREM2. In 5xFAD mice expressing wild-type human TREM2, sTREM2 was found bound to the amyloid plaques (Song et al., 2018), consistent with sTREM2 having a role in regulating plaques. Note that the ability of sTREM2 to block Aβ aggregation and to disaggregate Aβ, might be shared with full-length TREM2, as they both bind Aβ oligomers (Vilalta et al., 2021), but this has not been tested. Humans (and mice) with heterozygous R47H TREM2 have more fibrous plaques with more neuritic pathology (Yuan et al., 2016), which again might be explained by either R47H sTREM2 promoting Aβ fibrillation, or by reduced microglial phagocytosis of plaques.

## Evidence That sTREM2 is Protective Against AD in Humans

CSF levels of sTREM2 fall significantly in early pre-symptomatic stages prior to AD diagnosis (when amyloid is aggregating), but rise during mild cognitive impairment (MCI) and AD (when tau is aggregating), and fall again during the dementia stages of AD (Heslegrave et al., 2016; Piccio et al., 2016; Suárez-Calvet et al., 2016, 2019; Bekris et al., 2018; Liu et al., 2018; Nordengen et al., 2019; Rauchmann et al., 2019; Ma et al., 2020). People with higher CSF levels of sTREM2 progress more slowly through MCI and AD, in terms of memory loss, clinical score and brain atrophy (Ewers et al., 2019, 2020; Edwin et al., 2020; Franzmeier et al., 2020). And this apparent protective effect of sTREM2 correlated with reduced amyloid and Tau aggregation measured by PET (Ewers et al., 2020), consistent with sTREM2 reducing amyloid aggregation and pathology.

However, these apparent protective effect of high sTREM2 has been attributed to full-length TREM2, rather than sTREM2, on the untested assumption that high sTREM2 levels indicates high TREM2 levels, as a result of constant shedding. However, if elevated sTREM2 results from elevated shedding, which is for example induced by oligomeric Aβ (Vilalta et al., 2021), then this will reduce full-length TREM2. Thus, elevated levels of sTREM2 do not necessarily indicate that levels of full-length TREM2 are elevated, and the apparent protective effect of sTREM2 against AD may be more simply explained by sTREM2 itself being protective.

GWAS studies of gene variants that affect the CSF levels of sTREM2 identified the membrane-spanning 4-domains superfamily A (*MS4A*) gene cluster as key determinants of sTREM2 levels in CSF (Piccio et al., 2016; Deming et al., 2019; Hou et al., 2019). This gene region had previously been linked to AD risk (Naj et al., 2011). For example, rs1582763 increased brain expression of *MS4A4A* and *MS4A6A* genes, increased sTREM2 levels in CSF, reduced AD risk and increased age of AD diagnosis. While rs6591561 resulted in a loss-of-function *MS4A4A*, reduced CSF sTREM2 levels, increased AD risk and reduced age at AD onset (Deming et al., 2019). MS4A4A and TREM2 were found to colocalize at the plasma membrane, and overexpression of *MS4A4A* increased sTREM2 levels, whilst silencing of *MS4A4A* reduced sTREM2 levels (Deming et al., 2019). This suggests that MS4A4A may affect AD risk by promoting sTREM2 shedding, and if so, indicating that sTREM2, rather than full-length TREM2 is protective against AD. However, further work is required to establish whether MS4A4A directly affects sTREM2 shedding.

## Evidence Against The Hypothesis That sTREM2 Protects

One piece of evidence potentially contradicting a protective role of sTREM2 in AD, is that the H157Y mutation of TREM2 expressed in cells significantly increased sTREM2 shedding relative to wild-type TREM2, resulting in increased sTREM2 and decreased full-length TREM2, but is associated with increased AD risk (Schlepckow et al., 2017; Thornton et al., 2017). This suggests that the increased AD risk associated with the H157Y mutation is due to decreased full-length TREM2 or increased sTREM2, contradicting the hypothesis that sTREM2 is protective against AD. However, the H157Y mutation only increased shedding by about 50%, and this was from HEK293 cells (Schlepckow et al., 2017; Thornton et al., 2017), so it may be difficult to extrapolate to sTREM2 levels in human brains. Additionally, the H157Y mutation would constitute the C-terminal of sTREM2, and might affect its properties, such as its interactions with Aβ. Thus, it would be important to determine whether this mutation does indeed increase CSF levels of sTREM2 in humans, and whether H157Y sTREM2 has the same protective properties as wild-type sTREM2.

Other evidence potentially contradicting the hypothesis that sTREM2 protects against AD is the finding of Schlepckow et al. (2020) that an antibody binding to the ADAM cleavage site of TREM2 prevented sTREM2 release, but reduced plaques load in an amyloid mouse model. However, the antibody used directly activated TREM2 signaling, so the reduced plaque load may result from this signaling (Schlepckow et al., 2020). Additionally, the compaction of these plaques, neuritic pathology and memory loss were not tested in this model.

## Discussion

### Is TREM2 or sTREM2 Protective in Alzheimer's Disease?

It appears that either TREM2 or sTREM2 are protective in Alzheimer's disease, but which? TREM2 is thought to be protective by (i) recruiting and activating microglia into a protective state around amyloid plaques, and (ii) compacting amyloid plaques by phagocytosis of Aβ, preventing the plaques inducing neuritic pathology (Condello et al., 2015; Yuan et al., 2016; Keren-Shaul et al., 2017). Whereas, sTREM2 is thought to be protective by: (i) stimulating microglial recruitment, activation and phagocytosis of Aβ, and/or (ii) blocking and reversing Aβ aggregation, preventing neurotoxicity (Zhong et al., 2019; Vilalta et al., 2021). Thus, the putative protective effects of TREM2 and sTREM2 are complimentary rather than antagonistic, and potentially both may be protective against Alzheimer's disease. However, it is still important to verify that TREM2 and/or sTREM2 are in fact protective.

### Key Experiments to Determine Whether sTREM2 Is Protective Against AD

Some of evidence indicating that sTREM2 is protective against AD, may alternatively be interpreted as full-length TREM2 is protective. Thus, there is a need for experiments that distinguish between these possibilities, or directly show that sTREM2 is protective. The most direct way to show that is to add or express sTREM2 independent of full-length TREM2 and test whether this is protective in AD models. This has been done for a mouse amyloid model and found to be protective (Zhong et al., 2019), but this was relatively acute model, and it would be important to test this in other models, particularly more chronic and AD-relevant models. Within such models, it would be important to test whether sTREM2 can block Aβ aggregation, or disaggregate preformed plaques or oligomers. It would also be useful to know whether Aβ oligomers in AD CSF are significantly bound to sTREM2, and whether physiological levels of sTREM2 can disaggregate Aβ aggregation in CSF. Further, it would be worth knowing whether the different types of sTREM2 behave differently, including sTREM2 generated by ADAM and meprin β, or by alternative splicing, or H157Y and R62H sTREM2.

### Potential Treatment Strategies

Current strategies targeting TREM2 in AD have focused on agonistic antibodies to activate TREM2 with the aim of increasing microglial phagocytosis of amyloid plaques (Wang et al., 2020; Fassler et al., 2021). These antibodies will also bind sTREM2 and potentially block the protective effects of sTREM2 (Fassler et al., 2021). If sTREM2 is indeed more protective against AD than full-length TREM2, then antibodies that increased sTREM2 shedding might be beneficial, or other treatments designed to activate sTREM2 shedding e.g., by activating ADAM10 and ADAM17. Blocking sTREM2 degradation (e.g., by inhibiting meprin β) might increase sTREM2 levels without decreasing full-length TREM2. sTREM2 and sTREM2 fragments injected into the brain were protective in mouse models of AD (Zhong et al., 2019; Sheng et al., 2021), but may be difficult to deliver practically in humans. However, viral vectors expressing sTREM2 in the brain were protective in these mouse models of AD, and thus might be protective in humans with AD (Zhong et al., 2019).

## Author Contributions

GB wrote the article. PG-H reviewed and adjusted the article. Both authors were responsible for its content.

## Funding

This project has received funding from the Innovative Medicines Initiative 2 Joint Undertaking under Grant Agreement No. 115976. This Joint Undertaking receives support from the European Union's Horizon 2020 Research and Innovation Programme and EFPIA. PHStGH also received funding from the Canadian Institutes of Health Research (Foundation Grant and Canadian Consortium on Neurodegeneration in Aging Grant), Wellcome Trust Collaborative Award 203249/Z/16/Z, US Alzheimer's Society Zenith Grant ZEN-18-529769, and the Alzheimer's Society of Ontario Chair in Alzheimer's Disease Research.

## Conflict of Interest

The authors declare that the research was conducted in the absence of any commercial or financial relationships that could be construed as a potential conflict of interest.

## Publisher's Note

All claims expressed in this article are solely those of the authors and do not necessarily represent those of their affiliated organizations, or those of the publisher, the editors and the reviewers. Any product that may be evaluated in this article, or claim that may be made by its manufacturer, is not guaranteed or endorsed by the publisher.
